# An Infant Born to a Mother with Gestational Diabetes Presenting with 49,XXXXY Syndrome and Renal Agenesis-A Case Report

**DOI:** 10.4274/Jcrpe.764

**Published:** 2012-12-19

**Authors:** Dulika Sumathipala, Thilini Gamage, Bandula Wijesiriwardena, Rohan W. Jayasekara, Vajira H.W. Dissanayake

**Affiliations:** 1 Human Genetics Unit, Faculty of Medicine, University of Colombo, Colombo, Sri Lanka; 2 National Hospital of Sri Lanka, Colombo, Sri Lanka

**Keywords:** 49, XXXXY, chromosomal aneuploidy, gestational diabetes, renal agenesis

## Abstract

49,XXXXY is a rare sex chromosome polysomy with an incidence of 1 in 85 000 male births. It has a characteristic triad of mental retardation, skeletal malformation and hypogonadism. This is the first case report of a child with 49,XXXXY syndrome and renal agenesis. This child was referred for genetic testing at 14 years of age due to facial dysmorphism and hypergonadotropic hypogonadism. He had coarse facial features, cryptorchidism of the right testis, genu valgus deformities, and patent ductus arteriosus which are known associations of 49,XXXXY syndrome. He also had agenesis of the right kidney, hydronephrosis of the left kidney with hydroureter which is not a known association of 49,XXXXY syndrome. The patient was the offspring of a mother with gestational diabetes. There is a strong correlation between maternal diabetes and congenital anomalies, especially renal and cardiovascular anomalies. Additionally, it has been noted that gestational diabetes increases the incidence of chromosomal aneuploidies. The teratogenic effects of maternal diabetes during embryogenesis may be the causative factor for the final phenotype of 49,XXXXY syndrome and renal agenesis.

**Conflict of interest:**None declared.

## INTRODUCTION

Sex chromosome polysomy is varied, with 49,XXY or Klinefelter syndrome being the most common. 49,XXXXY was initially labeled as a “Klinefelter variant”. Yet, with the identification of a characteristic triad of mental retardation, skeletal malformation and hypogonadism, 49,XXXXY was categorized as a separate syndrome (1). The incidence is 1 in 85 000 male births (2). In Sri Lanka, this is the second detected case of 49,XXXXY, the first being detected in a baby boy with ambiguous genitalia (3). In this case report, we describe a patient diagnosed as 49,XXXXY and renal agenesis at age 14 years.

## CASE REPORTS

A 14-year-old boy was referred with hypergonadotropic hypogonadism and facial dysmorphism for genetic testing. He was the first born child to non-consanguineous parents. His two younger sisters have unremarkable medical histories. The pregnancy was complicated with gestational diabetes detected at 20 weeks of gestation and treated with subcutaneous insulin. The patient was delivered at 37 weeks gestation by lower segment caesarian section and his birth weight was 3.32 kg (97^th^ percentile). He had delayed motor and speech development. Walking without aid was achieved at 3 years and speech at 4 years of age. An assessment of intelligence was done at age 14 years using the test of non-verbal intelligence 3. A raw score of 10 was obtained which placed him at age 5 to 6 years in development. The patient had a history of a congenital heart defect reported as patent ductus arteriosus. He also was reported to have a seizure disorder and to receive antiepileptics.

On examination, the patient was 175 cm (97^th^ percentile) tall and weighed 66 kg (97^th^ percentile). He had a characteristic coarse facial appearance with prognathism, hypertelorism, epicanthal folds, upslanting palpebral fissures and a broad nasal bridge. He had cryptorchidism of the right testis with a hypoplastic scrotal sac. Genu valgus deformity of the lower limbs with multiple surgical corrections was also present.

A non-contrast computed tomographic scan of the brain revealed no abnormalities. An ultrasound scan of the abdomen revealed a solitary left kidney with gross hydronephrosis and hydroureter.

The patient had an elevated luteinizing hormone level (97.7 mIU/mL, normal range: 1.7-8.6 mIU/mL). Follicle-stimulating hormone level was also elevated (135 mIU/mL, normal range: 0.7-11.4 mIU/mL). Testosterone level was low (0.5 ng/mL, normal median 3.6 ng/mL). Based on these findings, a diagnosis of hypergonadotropic hypogonadism was reached.

A G-banding procedure was used for the analysis of metaphase chromosomes on peripheral blood cell cultures of the patient. Twenty metaphases were analyzed microscopically.

**Figure 1:**
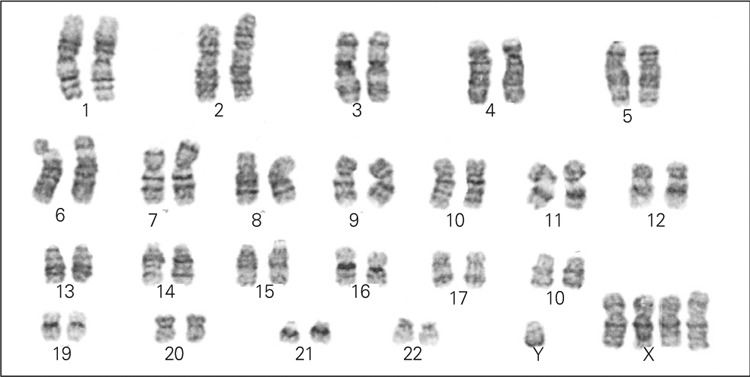
G-banded karyotype of the patient showing 49,XXXXY aneuploidy

Chromosome culture and karyotyping showed a karyotype of 49,XXXXY in all spreads analyzed (Figure 1).

## DISCUSSION

49,XXXXY syndrome is a rare chromosomal abnormality and is believed to occur during maternal non-disjunction both in meiosis I and II (4). The study of the role of diabetes mellitus in the etiology of chromosomal aneuploidies has shown that offspring of mothers with gestational diabetes have a crude prevalence of chromosomal defects that are twice as high as those seen in offspring of women without gestational diabetes mellitus (1,5). This has brought forth the theory that infants of mothers with gestational diabetes mellitus may have underlying biochemical changes that induce chromosomal non-disjunction and aneuploidies.

Classical features described in 49,XXXXY include skeletal malformations, mental retardation and hypogonadism. Facial features include prognathism, hypertelorism, broad nasal bridge, epicanthal folds, and a short broad neck. Cardiac defects are present in 15-20% of the patients, with patent ductus arteriosus being the most common (1). Skeletal abnormalities are frequent and present with radioulnar synostosis, genu valgus, pes cavus, and hyperextensible joints. Genitalia are usually hypoplastic and cryptorchidism is present. The IQ of patients with 49,XXXXY range from 60 to 20; in males, an extra X chromosome which leads to a reduction in IQ levels by 15 points has been reported (1). The coarse facial features, genu valgus deformity, cryptorchidism and developmental delay in our patient are well associated features of 49,XXXXY. However, to our knowledge, this is the first case of 49,XXXXY syndrome described with renal agenesis.

There is a strong correlation between maternal diabetes and congenital anomalies, especially renal and cardiovascular congenital anomalies (6). The renal agenesis seen in this patient is probably due to maternal diabetes. In newborns with 49,XXXXY syndrome, below-average lengths and weights are reported at birth (7). Yet, this patient had an above-average birth weight, which is in line with the fetal macrosomia seen in gestational diabetes. This finding strengthens the assumption that both the excess genetic material from the multiple X chromosomes as well as the teratogenic effects of maternal diabetes during embryogenesis contributed to the final phenotype of the patient. Therefore, it can be concluded that this patient had a dual teratogenic phenotype with genetic and biochemical agents having contributed to the ultimate phenotype.

Therapeutic options for 49,XXXXY are currently being researched. A study by Samango-Sprouse et al (8) investigates the role of early androgen treatment in 49,XXXXY. They question whether an early course of androgen treatment (three injections of testosterone enanthate, 25 mg) could have a positive impact on any domains of neurodevelopmental function. Initial findings have shown significant positive treatment effect in gestural communication and vocabulary development. These improvements in language development are consistent with smaller earlier studies in adult men with 47, XXY, which showed improved verbal skills when testosterone treatment was given (9,10).

In the presence of maternal gestational diabetes, it should be noted that offspring are at an increased risk for chromosomal aneuploidy as well as for congenital anomalies. If phenotypic variations are detected, chromosomal assessment should be included in further management.

Therapies for genetic conditions are establishing themselves through research. For such treatment options to become effective, early identification and referral for treatment is needed. Hence, karyotyping is warranted in the presence of facial dysmorphism or other somatic abnormalities to exclude underlying sex chromosomal aneuploidy disorders such as 49,XXXXY syndrome.

**Consent**

Proxy consent was obtained from the patient’s father for publication of this case report and accompanying images.

**Acknowledgements**

We would like to acknowledge the contribution made by Prof Hemamali Perera, Professor in Psychological Medicine, University of Colombo, for performing the IQ test for this child.
